# The mRNA-Binding Protein KSRP Limits the Inflammatory Response of Macrophages

**DOI:** 10.3390/ijms25073884

**Published:** 2024-03-30

**Authors:** Vanessa Bolduan, Kim-Alicia Palzer, Christoph Hieber, Jenny Schunke, Michael Fichter, Paul Schneider, Stephan Grabbe, Andrea Pautz, Matthias Bros

**Affiliations:** 1 Department of Dermatology, University Medical Center, Langenbeckstr. 1, 55131 Mainz, Germany; 2 Department of Pharmacology, University Medical Center, Langenbeckstr. 1, 55131 Mainz, Germanypautz@uni-mainz.de (A.P.); 3 Max Planck Institute for Polymer Research, Ackermannweg 10, 55128 Mainz, Germany

**Keywords:** RNA-binding proteins, cytokines, chemokines, KSRP, macrophages, LPS-induced sepsis

## Abstract

KH-type splicing regulatory protein (KSRP) is a single-stranded nucleic acid-binding protein with multiple functions. It is known to bind AU-rich motifs within the 3′-untranslated region of mRNA species, which in many cases encode dynamically regulated proteins like cytokines. In the present study, we investigated the role of KSRP for the immunophenotype of macrophages using bone marrow-derived macrophages (BMDM) from wild-type (WT) and KSRP^−/−^ mice. RNA sequencing revealed that KSRP^−/−^ BMDM displayed significantly higher mRNA expression levels of genes involved in inflammatory and immune responses, particularly type I interferon responses, following LPS stimulation. In line, time kinetics studies revealed increased levels of interferon-γ (IFN-γ), interleukin (IL)-1β and IL-6 mRNA in KSRP^−/−^ macrophages after 6 h subsequent to LPS stimulation as compared to WT cultures. At the protein level, KSRP^−/−^ BMDM displayed higher levels of these cytokines after overnight stimulation. Matching results were observed for primary peritoneal macrophages of KSRP^−/−^ mice. These showed higher IL-6, tumor necrosis factor-α (TNF-α), C-X-C motif chemokine 1 (CXCL1) and CC-chemokine ligand 5 (CCL5) protein levels in response to LPS stimulation than the WT controls. As macrophages play a key role in sepsis, the in vivo relevance of KSRP deficiency for cytokine/chemokine production was analyzed in an acute inflammation model. In agreement with our in vitro findings, KSRP-deficient animals showed higher cytokine production upon LPS administration in comparison to WT mice. Taken together, these findings demonstrate that KSRP constitutes an important negative regulator of cytokine expression in macrophages.

## 1. Introduction

Macrophages are present in all tissues of the body. When activated by pathogens or inflammation, macrophages exert various cytotoxic functions such as phagocytosis and attract immune cells to the site of inflammation by releasing cytokines and chemokines [1]. These properties make them a crucial component of the immune system. Activated macrophages secrete pro-inflammatory cytokines, such as tumor necrosis factor-α (TNF-α), interleukin (IL)-1β, and IL-6, as well as other pro-inflammatory mediators like nitric oxide (NO) and prostaglandins [2]. Moreover, macrophages serve as antigen-presenting cells and activate antigen-specific T cells [3].

Gene expression is orchestrated at the transcriptional level by transcription factors and long non-coding RNAs (lncRNA) that bind to cognate recognition sites within the gene promoter and enhancer/silencer regions, thereby regulating gene transcription [4]. In addition, genes which require dynamic regulation in response to stimuli are regulated at the post-transcriptional level [5]. In this regard, micro(mi)RNAs bind sequence-complementary sequence stretches within their target mRNA to limit mRNA stability and translational efficacy, respectively [6]. In addition to miRNA, RNA-binding proteins (RBP) also exert post-transcriptional gene regulation [7]. One class of RBP preferably engages AU-rich elements (ARE), which are mainly located in the 3′ untranslated region (UTR) of mRNA species [8]. AREs are the most prevalent recruiting motifs for RBP [9] and contain one or more core pentamers (e.g., AUUUA), often arranged in tandem repeats, and preferably located in a U-rich region [10]. Generally, AREs are characteristic of mRNAs encoding short-lived proteins, such as pro-inflammatory cytokines, transcription or growth factors [11]. Upon binding to their target sequences, ARE-RBPs either enhance (e.g., human antigen R, HuR) or limit (e.g., Tristetraprolin, TTP) mRNA stability and translation efficacy (e.g., T-Cell-Restricted Intracellular Antigen-1, TIA-1), respectively [9,12]. By now, an increasing number of studies have revealed the important role of RBP regarding the regulation of immune cell phenotypes [13,14].

KSRP (K homology [KH]-type splicing protein) is a ubiquitously expressed single-stranded nucleic acid-binding protein that has been reported to act as an mRNA stability-limiting RBP [15]. Additionally, it functions as a transcription factor and a maturation factor for various miRNA species [12]. So far, little is known about the cell type-specific function of KSRP in the immune system. In accordance with its central role in ARE-mediated decay of pro-inflammatory mediators, KSRP-deficient mice displaying a complete knockout in all tissues have been shown to present stronger anti-viral responses upon infection accompanied by elevated production of type I interferons [16]. Accordingly, KSRP has been considered an important negative regulator of inflammatory immune responses. In this regard, KSRP has been reported to limit cytokine production of activated immune cells, since it promoted decay of the according mRNA, as observed in cell culture experiments [17] and when assaying primary cells isolated from KSRP^−/−^ mice [13,18,19]. Further, we reported an increased production of Th2-associated cytokines by polyclonal stimulated KSRP-deficient T cells [20].

This study aimed to delineate the role of KSRP as a regulator of the immune phenotype of macrophages. We show that KSRP-deficient macrophages are characterized by a stronger inflammatory response to LPS stimulation in vitro and in vivo, suggesting that KSRP is an important negative regulator for pro-inflammatory cytokine expression under disease conditions.

## 2. Results

To date, little is known about the role of the RNA-binding protein KSRP on the gene regulation of innate immune cells, such as macrophages. Since KSRP seems to play a crucial role by regulating the mRNA expression of pro-inflammatory cytokines, we hypothesized that KSRP-deficient macrophages may show a stronger inflammatory response in response to LPS stimulation.

### 2.1. KSRP^−/−^ Mice Displayed Higher mRNA Expression Levels of Stimulation-Induced Cytokines

First, we analyzed bone marrow-derived macrophages (BMDM) under basal conditions and in response to LPS stimulation by flow cytometry. No differences were observed between WT and KSRP^−^^/−^ BMDM under basal conditions or in response to LPS stimulation with regard to BMDM cell numbers and activation marker expression (CD80, CD86, MHCII) (Figure S1). Furthermore, no genotype-dependent differences in metabolism were found in either condition (Figure S2).

**Figure 1:**
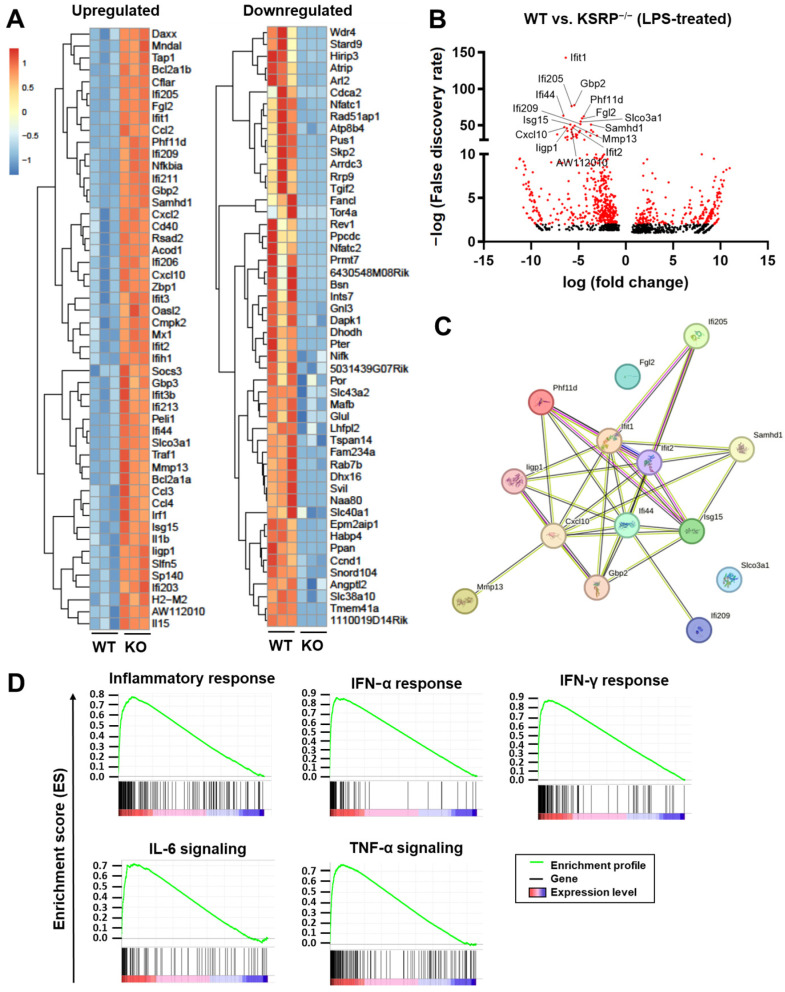
KSRP deficiency in BMDM results in upregulation of genes involved in inflammatory response, like IFN-α, IFN-γ, IL-6 and TNF-α signaling. LPS-stimulated BMDMs (WT and KSRP^−/−^, each n = 3) were subjected to RNA-seq analysis. (**A**) Heatmap representation of the top 50 significantly upregulated (left panel) and significantly downregulated (right panel) genes in WT versus KSRP^−/−^ BMDM (hierarchical clustering). The color legend denotes the level of gene expression (low: blue; high: red). (**B**) Volcano plot of significant (*p* < 0.1) quantified mRNA species. Significantly regulated genes (BH-adjusted *p* < 0.05 and log2 (fold-change) > 2) are given in red. The top 15 genes are named. (**C**) Results from analysis using STRING Database with the Top 15 upregulated gene in KSRP^−/−^ mice. (**D**) Gene set enrichment plots of significantly regulated pathways (BH-adjusted *p* < 0.05).

To obtain a broader view on KSRP-mediated transcriptional changes we subjected BMDM to RNA sequencing. Under basal conditions, KSRP-deficient BMDM were characterized by differential regulation of a total of 142 genes (up: 86; down: 56) compared to the WT group, the top 10 regulated genes are shown in Figure S3. For instance, the transcription factor *Zfp384* (zinc finger protein 384), limiting cytokine/chemokine gene transcription in macrophages in response to viral infection [21], or *Zbtb2* (zinc finger and BTB domain containing 2) inhibiting NF-κB activation [22] were upregulated in KSRP-deficient BMDM (Table S1). To assess the impact of KSRP on gene expression of pre-stimulated macrophages, BMDMs were treated with LPS as the most potent TLR4 ligand [23]. After 6 h of stimulation, BMDMs lacking KSRP expressed 951 genes to higher and 652 genes to lower extent compared to the corresponding control group (WT). Figure 1A shows the top 50 up- or downregulated genes. Interestingly, protein interaction analysis using STRING Database revealed that the top 15 upregulated genes in KSRP^−/−^ mice (Figure 1B), are interlinked and contribute to the regulation of innate immune response, particularly with regard to the cellular response to type I interferons (Figure 1C). Gene set enrichment analysis (GESA) indicated higher expression of many genes involved in the inflammatory response in KSRP-deficient BMDM (Figure 1D).

### 2.2. KSRP^−/−^ Mice Show Higher Protein Expression of IFN-γ, IL-1β and IL-6 in Response to Stimulation

**Figure 2:**
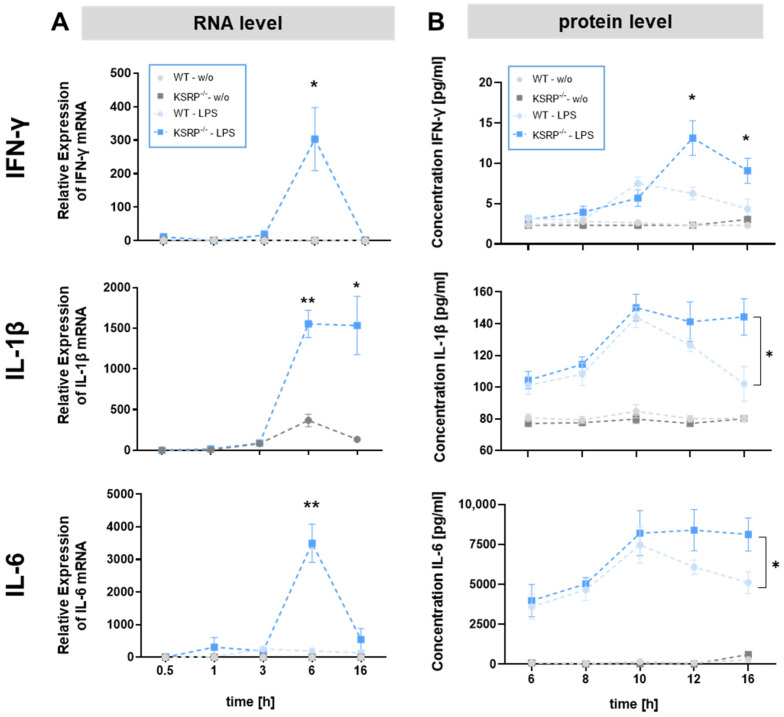
BMDM of KSRP^−/−^ animals display higher mRNA expression and protein expression of IFN-γ, IL-1β and IL-6 in response to stimulation. (**A**) To analyze the mRNA expression of different immune relevant genes in cells of KSRP^−/−^ or WT animals, BMDM were left untreated or were stimulated with 1 µg/µL LPS for different time periods (0.5 h, 1 h, 3 h, 6 h and 16 h). Subsequently, we prepared total RNA by homogenizing the sample in RLT plus lysis buffer and isolated the RNA using the RNeasy Plus Mini Kit. cDNA was synthesized by applying the iScript kit. Specific mRNA expression was measured using the qRT-PCR method and normalized to β2M mRNA expression. Shown are the mean ± SEM of n = 3–4 analyses (** *p* < 0.01, * *p* < 0.05; versus untreated WT cells; two-tailed Mann–Whitney test). (**B**) To analyze the protein expression of different immune-relevant genes in cells of KSRP^−/−^ or WT animals, BMDM were left untreated or were stimulated with 1 µg/µL LPS for different time periods (6 h, 8 h, 10 h, 12 h and 16 h). Supernatants of BMDM were collected and cytokine levels were determined using the Anti-Virus-Response LegendPlex Kit from BioLegend (San Diego, CA, USA). Shown are the means ± SEM of n = 7–8 analyses (* *p* < 0.05; versus untreated cells; two-tailed Mann–Whitney test).

Time kinetics studies confirmed higher levels of IFN-γ and IL-6 mRNA in KSRP^−/−^ versus WT macrophages after 6 h and of IL-1β mRNA after 6 h and 16 h (Figure 2A). In agreement, KSRP^−/−^ BMDM displayed higher IFN-γ levels after 12 h and 16 h, while IL-1β and IL-6 showed higher protein levels after 16 h (Figure 2B).

### 2.3. KSRP Binds Directly to IL-1β mRNA

**Figure 3:**
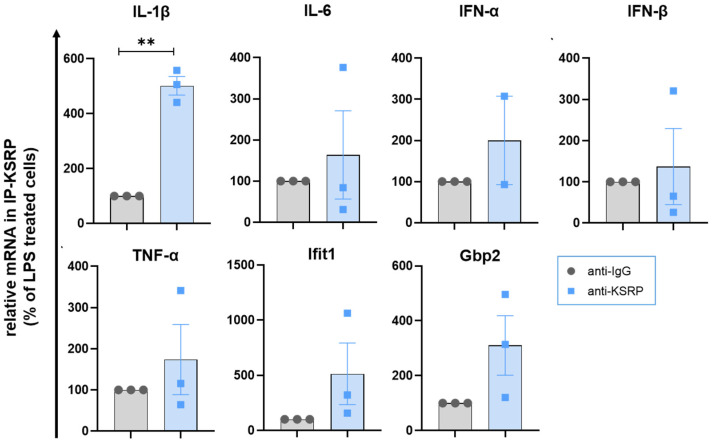
KSRP may regulates IL-1β mRNA stability by direct binding to its mRNA. WT BMDM were incubated with LPS (1 µg/mL) to induce pro-inflammatory chemo/cytokines for 6 h. After lysis of the cells, RNAs that bound to KSRP were immunoprecipitated with a specific antibody. To standardize the RNAs for the subsequent analyses, 1 ng of in vitro transcribed luciferase RNA was added to each sample. The RNA was purified, transcribed into cDNA and mRNA real time-PCR was used to determine the mRNA quantity of IL-1β, IL-6, IFN-α, IFN-β, TNF-α, Ifit1, Gbp2 and luciferase serving as a control for normalization. Shown are the means ± SEM and individual data points for each animal (n = 2–3 analyses) of the relative mRNA amounts bound to KSRP in relation to IgG controls (100%) (** *p* < 0.01; one-sample *t*-test).

To consider whether KSRP directly binds to the mRNAs of genes identified as stronger upregulated in stimulated KSRP-deficient versus WT BMDM, we used RNA immunoprecipitation. As KSRP is a multifunctional protein involved in various levels of gene regulation, this approach allowed us to investigate its role in mRNA regulation. KSRP directly binds to the mRNA of IL-1β, while the other examined mRNAs (IL-6, IFN-α, IFN-β, TNF-α, Ifit1, Gpb2) may not exhibit such binding (Figure 3). This indicates that IL-1b mRNA may directly regulated by KSRP.

### 2.4. KSRP^−/−^ Primary Macrophages from the Peritoneum Produce Higher Levels of Pro-Inflammatory Cytokines in Response to Stimulation

**Figure 4:**
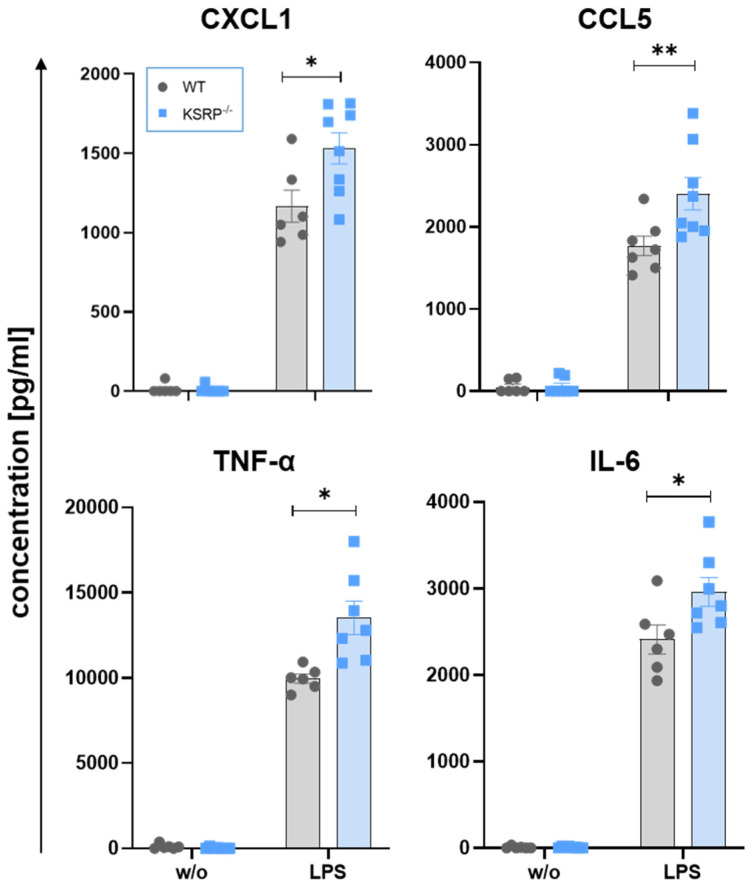
Inactivation of the KSRP gene enhances CXCL1, CCL5 (RANTES), TNF-α and IL-6 protein expression in murine peritoneal cells. Peritoneal cells were isolated from WT and KSRP^−/−^ mice. Adherent cells (mostly monocytes/macrophages) were used for the experiments. Adherent peritoneal cells were incubated with LPS (1 µg/mL) to induce pro-inflammatory chemo/cytokines. After 6 h supernatant was used for analysis using the Anti-Virus-Response LegendPlex- Kit from BioLegend. Shown are the means ± SEM of n = 6–8 analyses (* *p* < 0.05; ** *p* < 0.01 versus untreated cells; two-tailed Mann–Whitney test).

Bollmann et al. observed higher mRNA expression of chemokine (C-X-C motif) ligand (CXCL)1, inducible nitric oxide synthase and TNF-α in response to stimulation with LPS in adherent primary KSRP^−/−^ peritoneal cells compared to WT cells [18]. Therefore, we asked if expression of other (pro-inflammatory) cytokines was affected by KSRP deficiency as well. Hence, we also assessed primary macrophage-enriched cell suspensions obtained by peritoneal lavage and analyzed protein production of these cells. Regarding KSRP^−/−^ peritoneal cells we detected an enhanced production of pro-inflammatory cytokines, such as CXCL1, CCL5, TNF-α and IL-6 (Figure 4), whereas expression levels of other mediator remained unaltered.

### 2.5. KSRP^−/−^ Display Tendencies to Higher Proinflammatory Cytokine Production in LPS-Induced Sepsis

**Figure 5:**
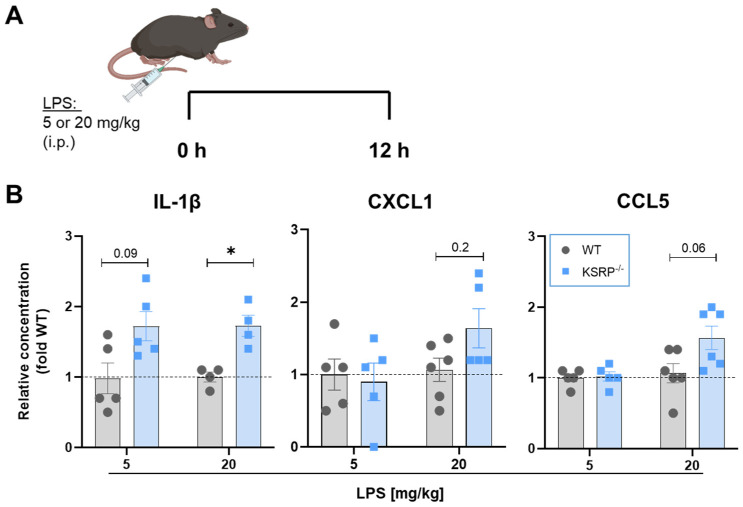
Inactivation of the KSRP gene increased cytokine production after treatment with 5 or 20 mg/kg LPS. (**A**) WT or KSRP^−/−^ mice were treated with 5 or 20 mg/kg LPS i.p. for 12h. Created with BioRender.com. (**B**) Analysis of different pro-inflammatory cytokines involved in sepsis progression. Shown are the means ± SEM of n = 4–6 analyses (* *p* < 0.05 versus WT cytokine expression; two-tailed Mann–Whitney test).

Our results indicate, that KSRP may act as an important negative regulator of inflammatory responses of macrophages in vitro. Due to the pronounced role of macrophages in sepsis, we further analyzed the effect of KSRP deficiency in an in vivo LPS-induced acute inflammation model focusing on early KSRP-dependent effects. To this end, mice were injected intraperitoneally (i.p.) with LPS at sub-lethal doses to stimulate pro-inflammatory cytokine expression [24] (Figure 5A). 12 h after injecting 5 mg/kg LPS i.p., we obtained some effects for IL-1β in KSRP-deficient mice, however below significance. After injection of high dose LPS (20 mg/kg), sera analyses revealed a significant increase in IL-1β production in KSRP-deficient mice compared to WT mice (Figure 5B). A similar trend was found for the chemokines CXCL1 and CCL5 in response to the higher LPS dose (Figure 5B).

In summary, these findings demonstrate that a deficiency of the RNA-binding protein KSRP in macrophages leads to a more rapid and increased production of pro-inflammatory cytokines in response to LPS stimulation. Therefore, KSRP constitutes an important negative regulator of cytokine production.

## 3. Discussion

Macrophages are innate immune cells that reside throughout the body. On the one hand they exert potent pathogen-killing activity by various mechanisms and on the other hand act as antigen-presenting cells, thereby inducing antigen-specific T cell responses [25]. To date, several RBPs like TTP, HuR, TIAR have been shown to influence the immunophenotype of macrophages [26,27,28,29]. By now, the multifunctional RBP KSRP has been identified as a general negative regulator of inflammatory immune responses by limiting cytokine production of activated immune cells via promoting decay [17,19,30] or inhibiting translation [31] of target mRNAs. Both functions are conferred by direct binding of KSRP to ARE within its target mRNA. However, KSRP may also act in an indirect manner by mediating the maturation of miRNA species, which in turn inhibit gene expression [32,33]. Due to the pronounced role of macrophages in innate and adaptive immune responses, we asked for the role of KSRP in shaping the immunophenotype of primary macrophages. We show that KSRP limits the inflammatory response of macrophages as evidenced by increased expression of inflammatory mediators in KSRP-deficient macrophages in response to stimulation in vitro. In line, KSRP^−/−^ mice displayed elevated cytokine production in an acute inflammation model known to be mediated in large part by activated macrophages.

No major differences in the frequency of macrophages in the spleen and liver of KSRP^−/−^ versus WT mice under homeostatic conditions were observed. Further, we noted no genotype-dependent differences in the expression of surface activation markers of macrophages under basal conditions or in response to LPS stimulation (Figure S1). Similarly, KSRP deficiency did not affect the glycolytic activity of unstimulated and stimulated macrophages, respectively (Figure S2). However, RNA sequencing analysis of KSRP-deficient BMDM under basal conditions showed, e.g., upregulation of *Zfp384*, which limits cytokine responses at the transcriptional level [21] or *Zbtb2* (zinc finger and BTB domain containing 2) inhibiting NF-κB activation [22]. These findings suggest that KSRP-deficiency under basal conditions may be balanced in part by the upregulation of other immune response limiting genes. However, this assumption needs to be verified in further experiments.

RNA sequencing after 6 h of LPS stimulation revealed higher mRNA expression levels of genes involved in inflammatory responses related to IL-6 or TNF-α signaling in KSRP-deficient BMDM. Interestingly, the top 15 upregulated genes in KSRP^−/−^ BMDM are interlinked and contribute to the cellular response of type I interferons. In line, Lin et al. demonstrated that KSRP is a crucial negative regulator of type I IFN gene expression at the post-transcriptional level by interacting with the 3′-UTRs of these mRNAs, as shown for mouse embryonic fibroblasts of KSRP^−/−^ mice [16]. In our study, time kinetics studies revealed higher mRNA and protein levels of IFN-γ, IL-1β, and IL-6 after LPS stimulation of KSRP-deficient BMDM. To evaluate our observations, we analyzed also primary peritoneal cells that are enriched in macrophages. In accordance with our findings on BMDM, LPS stimulation resulted in a significant upregulation of IL6, TNF-α, CXCL1 (KC) and CCL5 (RANTES) expression in KSRP^−/−^ peritoneal macrophages.

In agreement, previous studies have shown that KSRP regulates the expression of several pro-inflammatory mediators, including IL-1β [33,34], IL-6 [31,35], CCL5 [33], and TNF-α [31,34,35]. The results of our IP studies suggest direct binding of KSRP to LPS-induced IL-1β mRNA in BMDM, while IL-6, TNF-α, IFN-γ, IFN-α and IFN-β mRNAs were most probably regulated by KSRP in an indirect manner.

As outlined above, KSRP may regulate gene expression both in a direct and in an indirect manner. For instance, Li et al. observed stabilization of both IL-1β and TNF-α mRNA transcripts in LPS-stimulated astrocytes from KSRP^−/−^ mice [34]. Showing binding of KSRP to according mRNAs, suggesting a direct manner of KSRP regulation. As mentioned, Lin and coworkers already revealed regulation of *IFNA4* and *IFNB* mRNA stability by KSRP through interaction with the 3′-UTR [16]. Interestingly, Winzen et al., identified ~100 target mRNAs of KSRP, including IL-6, IL-8 and cyclooxygenase-2, whose expression levels were higher in KSRP-deficient cells [35]. However, mRNA degradation was only detected in 10% of all KSRP targets, which may be explained in part by the additional function of KSRP to inhibit mRNA translation. Dhamija and colleagues assessed the polysome profiles of cells with siRNA-mediated KSRP deficiency. Thereby KSRP was found to interact with the ARE of IL-6 mRNA and mediate its translational silencing [31].

In addition to direct interaction with mRNA resulting in decay or translational inhibition, KSRP may also regulate gene expression in an indirect manner. KSRP plays a crucial role in processing of a subset of miRNAs, particularly those containing a GC-rich stem-loop structure in their immature precursor transcript [36]. Among those are miR-155 [37], let-7a [38] and miR-129 [39], which exert important functions in the regulation of immune processes. Ruggiero and colleagues demonstrated miRNA-mediated degradation of IL-1β and CCL5 mRNA in a mouse macrophage cell line [33]. Also miRNA let-7a was reported to inhibit IL-6 expression in macrophages [40]. Taken together, KSRP regulates gene expression on various levels. In the nucleus, it acts as a transcription and splicing factor, while in the cytoplasm it mediates rapid decay of ARE-containing mRNAs or silences translation of mRNA. In addition to direct regulation of gene expression via post-transcriptional mechanisms, KSRP also acts in an indirect manner by promoting the maturation of a subset of miRNA species, which in turn affect expression of multiple genes. With regard to our data, these results suggest that KSRP may regulate expression of a given genes in a direct or indirect manner, depending on the cell type and experimental conditions. Further studies are necessary to elucidate the mode of KSRP-mediated regulation of its target genes in macrophages.

Sepsis is a multilayered disturbance of systemic immunologic homeostasis of inflammation and anti-inflammation [41]. In the course of sepsis, there is a systemic release of pro-inflammatory cytokines [42]. The increased expression of these factors is in part due to elevated activity of various transcription factors (e.g., NF-κB, STATs, AP-1) that confer enhanced gene expression. However, especially pro-inflammatory and immune cell function-modulating genes are also regulated at the post-transcriptional level by RBP [7].

In agreement with our in vitro findings, we demonstrate that KSRP^−/−^ mice displayed higher IL-1β levels in sera derived from LPS-treated mice and observed similar effects for other proinflammatory mediators albeit below statistical significance. This finding suggests that KSRP contributes to limit the extent of the cytokine storm in macrophages in the course of sepsis. Likewise, Liu et al. showed that the RBP AUF1 protected animals from endotoxic shock by reducing the expression of the pro-inflammatory cytokines TNF-α and IL-1β through mRNA degradation [24]. Similar to KSRP also deficiency of AUF1 under steady state conditions did not influence mouse development [24].

Our observations suggest that KSRP may be part of an immunological negative feedback loop limiting immune responses to prevent excessive damages of the host’s tissues both by direct mRNA binding but also via yet unknown indirect mechanisms. With regard to therapeutic approaches, KSRP activity could be temporarily enhanced to attenuate unwanted inflammatory immune responses, including sepsis. In this regard, we have already shown that resveratrol modulates KSRP mRNA-binding activity and thereby enhanced mRNA degradation, leading to anti-inflammatory effects [18]. Further studies are dedicated to elucidate the role of KSRP in this regard.

## 4. Materials and Methods

### 4.1. Mice

KSRP^+/−^ mice on C57BL/6 background [16] were bred and maintained in the Central Animal Facility of the Johannes Gutenberg University Mainz under specific pathogen-free conditions. KSRP^−/−^ (KO) and KSRP^+/+^ (WT) animals were obtained by mating KSRP^+/−^ animals. Genotyping of the animals was performed by polymerase chain reaction using the following primers: KSRP-wt-for GCGGGGAGAATGTGAAGG, KSRP^−/−^-for CTCCGCCTCCTCAGCTTG, and KSRP-wt/^−/−^ -rev GAGGCCCCTGGTTGAAGG. All animal procedures were performed in accordance with the institutional guidelines and have been approved by the National Investigation Office of Rhineland-Palatinate (approval ID: G17-1-061). Mice (8–14 weeks) of both sexes were used throughout all experiments.

### 4.2. Bone Marrow-Derived Macrophages (BMDM)

Bone marrow cells (4 × 10^5^/mL) were seeded in 12-well cell cluster plates (1 mL) (Greiner Bio-One, Kremsmünster, Austria) in IMDM-based culture medium containing 5% FCS (PAN-Biotech, Aidenbach, Germany), 2 mM L-glutamine, 100 U/mL penicillin, 100 µg/mL streptomycin, 50 µM ß-mercaptoethanol (all from Sigma-Aldrich, Deisenhofen, Germany), and supplemented with 10 ng/mL recombinant murine M-CSF (Miltenyi Biotec, Bergisch Gladbach, Germany). Culture media was replenished every 3 days of culture. BMDM were subjected to experiments on days 7–9 of culture unless indicated otherwise.

### 4.3. Isolation of Peritoenal Cells

Peritoneal cells were isolated from WT and KSRP^−/−^ mice using a 27G needle and ice-cold PBS supplemented with 3% FCS. Afterwards, cells were seeded in 24-well cell cluster plates (500 µL, 10 × 10^5^/mL) (Greiner Bio-One, Kremsmünster, Austria) in IMDM-base culture medium (see above) for 3 h at 5% CO_2_, 37 °C. Non-adherent cells were removed by gentle washing with pre-warmed PBS to enrich adherent monocytes/macrophages. Replicate wells were treated with LPS (1 µg/mL). After 6 h cell supernatant was collected and analyzed for cytokine contents (see Section 4.4).

### 4.4. Cytometric Bead Array

The cytokine secretion of cultivated and pretreated cells as well as cytokine levels in murine sera were quantified using a multiplex bead-based immunoassay (LEGENDplex Mouse Anti-Virus Response Panel; 13-plex, BioLegend, San Diego, CA, USA) in accordance with manufacturer’s instructions. Samples were measured in an Attune NxT flow cytometer (Thermo Fisher Scientific, Waltham, MA, USA). Data analysis was performed using LEGENDplex Qognit software (v8.0, BioLegend, San Diego, CA, USA).

### 4.5. Immunoprecipitation-qRT-PCR Assay

Immunoprecipitation-qRT-PCR assay was performed as described [19] with slight modifications. WT BMDM (d7) were treated with LPS (1 µg/mL) for 6 h to induce pro-inflammatory chemokines and cytokines. KSRP/RNA complexes were isolated using KSRP-specific antibody (pAB anti-KSRP, Novus Biologicals, Centennial, CO, USA) and a corresponding isotype control antibody (anti-mouse IgG, Sigma-Aldrich, Deisenhofen, Germany) in parallel reactions. Total RNA was purified using the GeneJET RNA Cleanup and Concentration Micro Kits (Qiagen, Hilden, Germany), transcribed into cDNA and qRT-PCR was used to determine quantities of IL-1β, IL-6, IFN-α, IFN-β, TNF-α, Ifit1 and Gbp2 mRNA.

### 4.6. Analysis of mRNA Expression in Cells or Tissues of KSRP^−/−^ or WT Animals

To analyze the mRNA expression of different immunorelevant genes in cells of KSRP^−/−^ or WT animals total RNA was isolated using the RNeasy Plus Mini Kit (Qiagen, Hilden, Germany). Total RNA was subjected to RNA sequencing (see Section 4.7) and real-time PCR (see Section 4.8) For real-time PCR cDNA was synthesized by applying the iScript kit (Bio-Rad, Munich, Germany).

### 4.7. RNA-Sequencing and Bioinformatical Analysis

A total of 4 × 10^5^ BMDM of WT and KSRP^−/−^ mice were cultured with LPS (1 µg/mL) for 6 h. RNA was purified with the RNeasy Plus Mini Kit according to the manufacturer’s protocol (Qiagen Hilden, Germany). NGS library prep was performed with Lexogen’s QuantSeq 3′mRNA-Seq Library Prep Kit FWD (Lexogen, Vienna, Austria) following Lexogen’s standard protocol with modifications for low Input RNA (≤10 ng) (015UG009V0260). Libraries were prepared with a starting amount of 6.9 ng and amplified in 22 PCR cycles. Libraries were profiled in a High Sensitivity DNA chip on a 2100 Bioanalyzer (Agilent technologies, Santa Clara, CA, USA) and quantified using the Qubit dsDNA HS Assay Kit, in a Qubit Flex Fluorometer (Life technologies, Carlsbad, CA, USA). All 12 samples were pooled together with 12 samples from another project in equimolar ratio and sequenced on 1 NextSeq 500 Highoutput Flowcell, SR (Illumina, Inc, San Diego, CA, USA)for 1 × 85 cycles plus 6 cycles for the index read. RNA-Seq reads were aligned with STAR aligner (v2.7.3a; [43]) to the GRCm39 genome with the parameters—outStd SAM—outMultimapperOrder Random—outSAMattributes NH HI AS nM MD—outFilterMismatchNmax 999—outFilterMismatchNoverReadLmax 0.04. Using the featureCounts program of subread software (v2.0.0; [44]) the primary alignments were assigned to exons with default parameters. The GENCODE mouse annotation release M26 was used in all the steps. Further, with only the uniquely mapped reads differential expression analysis was performed using the bioconductor release 3.14 ([45]) and DESeq2 (v1.34.0; [46]) where genes showing a Benjamini–Hochberg-adjusted FDR < 0.1 were considered differentially expressed. Results were illustrated using the R heatmap package. GraphPad Prism 9 (GraphPad Software Inc., San Diego, CA, USA) was used to create volcano plots of differentially expressed genes. The gene set enrichment analysis of normalized gene counts was performed using the GSEA 4.2.3 software (standard settings, gene set database: h.all.v7.5.1 [47,48]). A false discovery rate (FDR) *q*-value < 0.05 was considered statistically significant. STRING database (v12) was used to analyze protein-protein interaction networks. Transcriptome data have been deposited in the GEO database, accession number GSE261444.

### 4.8. Real-Time PCR

cDNA of differentially pretreated macrophages was used for real-time PCR using the following primers: IL-1β (5′-GCCCATCCTCTGTGACTCAT-3′, 5′-AGGCCACAGGTATTTTGTCG-3′), IL-6 (5′-CCGGAGAGGAGACTTCACAG-3′, 5′-CAGAATTGCCATTGCACAAC-3′), IFN-**γ** (5′-GCTTGCAGCTCTTCCTCAT-3′, 5′-GTCACCATCCTTTTGCCAGT-3′) and β2-Mikroglobulin (β2M) (5′-CGGCCTGTATGCTATCCAGA-3′, 5′-GGGTGAATTCAGTGTGAGCC-3′). All primers were obtained from Eurofins Scientific (Luxembourg City, Luxembourg). Reaction mixtures had a final volume of 20 µL and included 200 ng cDNA, 70 nM of each primer, and 12.5 µL of 2× primaQUANT Master Mix high ROX (Steinbrenner Laborsysteme, Wiesenbach, Germany). Each sample was tested in duplicate. Thermal cycling conditions were 95 °C for 10 min, 40 cycles of 95 °C for 15 s, and 60 °C for 1 min, followed by a melting curve stage of 95 °C for 15 s and 60 °C for 1 min using an ABI 7300 real-time PCR cycler (Applied Biosystems, Waltham, MA, USA). mRNA expression was normalized to β2M mRNA expression.

### 4.9. The LPS-Induced Sepsis Model

WT and KSRP^−/−^ mice were injected with LPS (5 or 20 mg/kg bodyweight). After 12 h, blood samples were collected, centrifuged at 10,000× *g* for 8 min and sera were used for cytokine detection (see Section 4.4).

### 4.10. Statistical Analysis

Statistical analysis was performed using GraphPad Prism Software v9.0 (GraphPad Software Inc., San Diego, CA, USA). Results were expressed as the mean ± standard error of the mean (SEM).

## Supplementary Materials

The following supporting information can be downloaded at: https://www.mdpi.com/article/10.3390/ijms25073884/s1.
