# Supplementary material for: The mRNA-Binding Protein KSRP Limits the Inflammatory Response of Macrophages
Source: Int J Mol Sci. 2024 Mar 30;25(7):3884. doi: 10.3390/ijms25073884 (PMC11011855; doi:10.3390/ijms25073884)
Supplement: Supplementary file 1 [file ijms-25-03884-s001.zip › ijms-2938820-supplementary.pdf]

International Journal of *Molecular Sciences*

MDPI

Supplementary Material

The mRNA-binding protein KSRP limits the inflammatory response of macrophages

Vanessa Bolduan[1], Kim-Alicia Palzer[2], Christoph Hieber[1], Jenny Schunke[1], Michael Fichter[1,3], Paul Schneider[1], Stephan Grabbe[1], Andrea Pautz[2], Matthias Bros[1,*]

[1] Department of Dermatology, University Medical Center, Mainz, Langenbeckstr. 1, 55131 Mainz, Germany
[2] Department of Pharmacology, University Medical Center, Mainz, Langenbeckstr. 1, 55131 Mainz, Germany
[3] Max Planck Institute for Polymer Research, Ackermannweg 10, 55128 Mainz, Germany
* Correspondence: mbros@uni-mainz.de

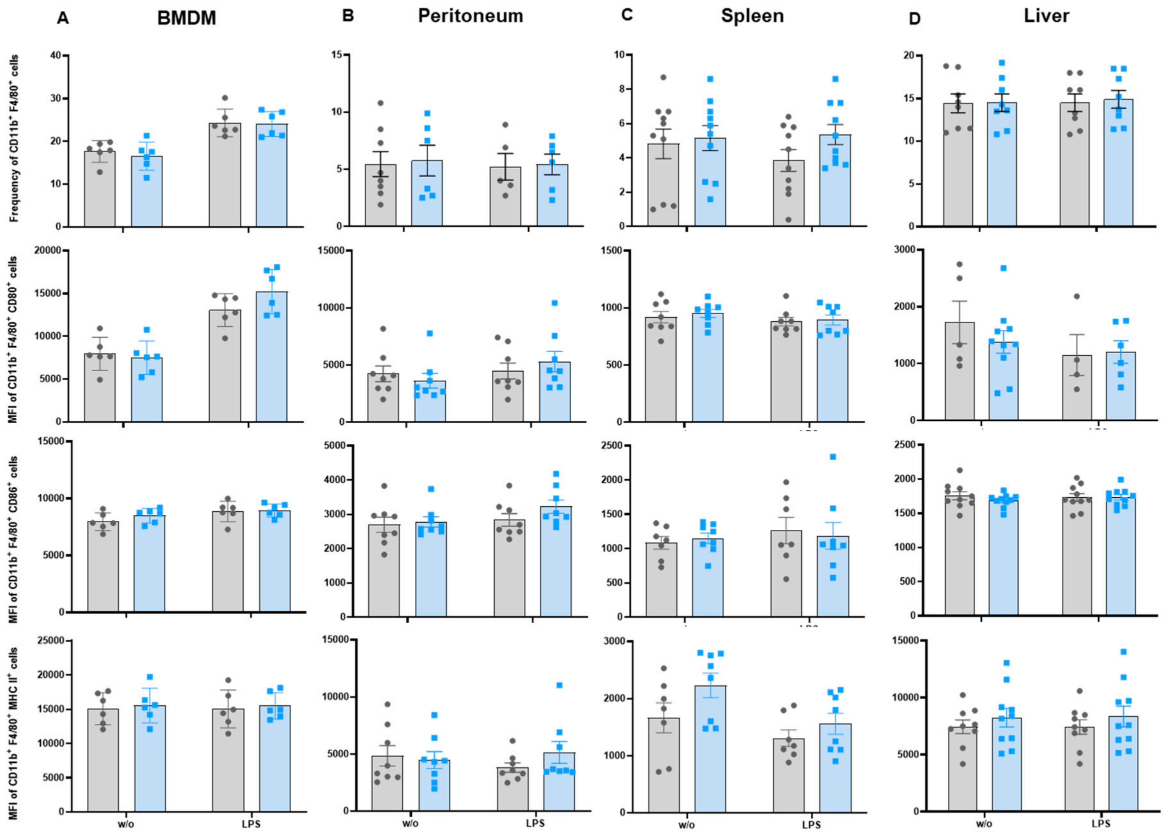

Fig. S1: KSRP mice show no differences concerning cell frequency and surface activation markers. Cells were isolated from the bone marrow and differentiated with 10 ng/ml M-CSF to BMDM or directly isolated from the different organs and unstimulaed (w/o) or stimulated (1 µg/µl LPS, 6h) used for flow cytometic staining. Analysis of Bone marrow-derived macrophages (BMDM) (A), Peritoneal macrophages (B), splenic macrophages (C) and liver macrophages (D) concerning macrophage freuqnecy and activation marker expression. and liver macrophages (D) concerning macrophage frequency and activation marker expression. Cells were stained for flow cytometric analysis as described earlier (Bednarczyk M et al., 2022.). To this regard, cells were incubated with FITC-conjugated anti-CD86 (GL-1) and anti-MHCII (M5/114.15.2), PerCP-eFluor710-conjugated anti-CD80 (16-10A1), APC-conjugated anti-F4/80 (BM8), eFluor450-conjugated anti-CD86 (GL-1), Super Bright 700-conjugated anti-CD11b (M1/70), PE-conjugated anti-CD80 (16-10A1) and PE-eFluor 610-

conjugated anti-MHCII (M5/114.15.2). Antibodies were purchased from BD Biosciences (Franklin Lakes, NJ, USA), Thermo Fisher (Scientific, Waltham, MA, USA) or BioLegend (San Diego, CA, USA).

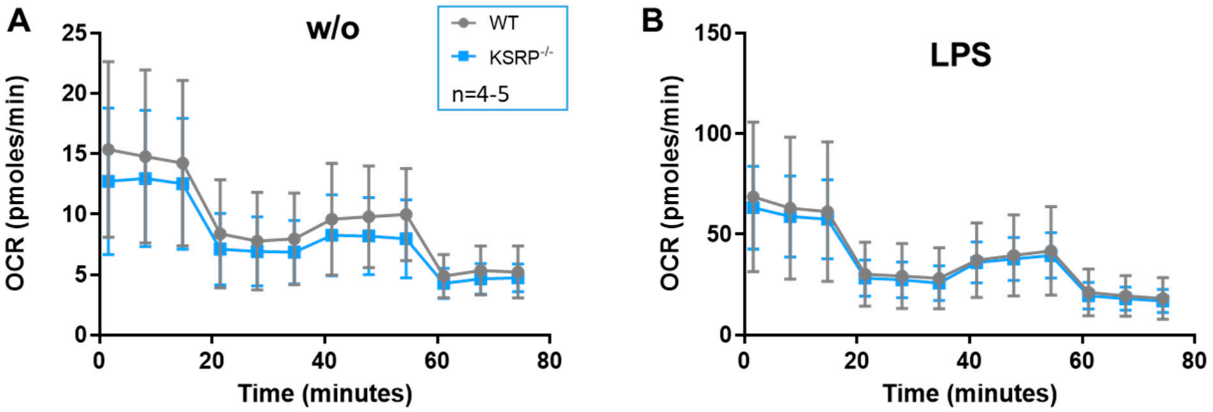

Fig. S2: KSRP BMDM show no differences concerning metabolic activity under basal conditons (A) and in response to LPS stimulation (B). Bone marrow progenitor cells were isolated from KSRP WT and KO mice and differentiated with M-CSF for 7 days to generate bone marrow-derived macrophages (BMDM). Seahorse Cell Mito Stress assays were performed according to the manufacturer's protocol and specifications for a Seahorse XFp Analyzer (Agilent, Santa Clara, CA, USA). In brief, the day before the assay, BMDM were plated at 30,000 cells per well in standard cell culture medium in Seahorse XFp Cell Culture Miniplates (Agilent, #103025-100). The following day, medium was exchanged for Seahorse assay medium consisting of XF RPMI (Seahorse XF RPMI medium, Agilent, #103576-100) supplemented with 1 mM pyruvate (Agilent, #103578-100), 2 mM glutamine (Agilent, #103579-100), and 10 mM glucose (Agilent, #103577-100). Cells were treated with 1 µg/µl LPS incubated for 6 h in a 37 °C 10% CO_2 incubator. Meanwhile, Seahorse sensor cartridges (Seahorse XFp FluxPak, Agilent, #103022-100) were loaded with 1.5 µM oligomycin, 1.0 µM FCCP, and 0.5 µM rotenone/antimycin A (Seahorse XFp Cell Mito Stress Test Kit, Agilent, #103010-100). Plates were placed into a Seahorse XFp Analyzer and the program "Seahorse XF Mito Stress Test" was ran.

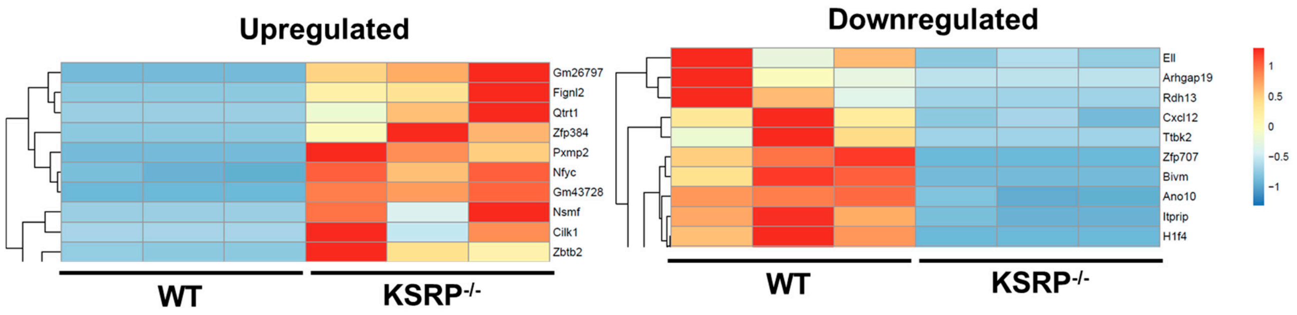

Fig. S3: Unstimulated BMDMs (WT and KSRP$^{-/-}$, each n=3) were subjected to RNA-seq analysis. Shown is a heatmap representation of the top 10 significantly downpregulated (left panel) and significantly upregulated (right panel) genes in WT versus KSRP$^{-/-}$ BMDM (hierarchical clustering). The color legend denotes the level of gene expression (low: blue; high: red).

Table S1: Top 10 up/down regulated genes under basal conditions of WT and KSRP$^{-/-}$ mice.

	Gene		Functions
	Symbol	Name	
Upregulated	Gm26797	predicted gene, 26797	-
	Fignl2	fidgetin like 2	enables microtubule-severing ATPase activity
	Qtrt1	queuine tRNA-ribosyltransferase catalytic subunit 1	catalytic subunit of tRNA-guanine transglycosylase. that confers tRNA modification by synthesizing 7-deazaguanosine queuosine (found in tRNAs that code for asparagine, aspartic acid, histidine and tyrosine)
	Zfp384	zinc finger protein 384	zinc-finger-containing transcription factor; shown to limit cytokine/chemokine expression in response to viral infection (**PMID: 32152414**)
	Pxmp2	peroxisomal membrane protein 2	peroxisomal channel; regulates lipid metabolism
	Nfyc	nuclear transcription factor Y subunit gamma	subunit of transcription factor binding CCAAT motifs
	Gm43728	predicted gene, 43728	-
	Nsmf	NMDA receptor synaptonuclear signaling and neuronal migration factor	
	Cilk1	ciliogenesis associated kinase 1	serine/threonine protein kinase
	Zbtb2	zinc finger and BTB domain containing 2	transcription factor (silencer); inhibits NF-kB activation (**PMID: 25609694**)
Downregulated	Ell	elongation factor for RNA Polymerase II	part of transcription elongation factor complex
	Arhgap19	Rho GTPase activating protein 19	regulates small GTPase-mediated signal transduction
	Rdh13	retinol dehydrogenase 13	catalyzes reduction/oxidation of retinoids; protects mitochondria against oxidative stress
	Cxcl12	C-X-C motif chemokine ligand 12	ligand of G-protein-coupled chemokine (C-X-C motif) receptor 4; broad immunological role
	Ttbk2	tau tubulin kinase 2	serine-threonine kinase; phosphorylates tau and tubulin proteins
	Zfp707	zinc finger protein 707	transcription factor
	Bivm	basic, immunoglobulin-like variable motif containing	-
	Ano10	anoctamin 10	calcium-activated chloride channel
	Itprip	inositol 1,4,5-trisphosphate receptor interacting protein	enhances sensitivity of inositol 1,4,5-trisphosphate receptor to intracellular calcium signaling
	H1f4	H1.4 linker histone, cluster member	engages linker DNA between nucleosomes to compact chromatin
